# Changes in music-evoked emotion and ventral striatal functional connectivity after psilocybin therapy for depression

**DOI:** 10.1177/02698811221125354

**Published:** 2022-11-26

**Authors:** Melissa Shukuroglou, Leor Roseman, Matt Wall, David Nutt, Mendel Kaelen, Robin Carhart-Harris

**Affiliations:** 1 Independent Researcher; 2 Centre for Psychedelic Research, Department of Medicine, Imperial College London, UK; 3 Computational, Cognitive and Clinical Neuroscience Laboratory (C3NL), Department of Medicine, Imperial College London, UK; 4 Invicro, London, UK; 5 Clinical Psychopharmacology Unit, UCL, UK; 6 Wavepaths Ltd, London, UK

**Keywords:** Depression, functional connectivity, music, pleasure, psychedelic

## Abstract

**Background:**

Music listening is a staple and valued component of psychedelic therapy, and previous work has shown that psychedelics can acutely enhance music-evoked emotion.

**Aims:**

The present study sought to examine subjective responses to music before and after psilocybin therapy for treatment-resistant depression, while functional magnetic resonance imaging (fMRI) data was acquired.

**Methods:**

Nineteen patients with treatment-resistant depression received a low oral dose (10 mg) of psilocybin, and a high dose (25 mg) 1 week later. fMRI was performed 1 week prior to the first dosing session and 1 day after the second. Two scans were conducted on each day: one with music and one without. Visual analogue scale ratings of music-evoked ‘pleasure’ plus ratings of other evoked emotions (21-item Geneva Emotional Music Scale) were completed after each scan. Given its role in musical reward, the nucleus accumbens (NAc) was chosen as region of interest for functional connectivity (FC) analyses. Effects of drug (vs placebo) and music (vs no music) on subjective and FC outcomes were assessed. Anhedonia symptoms were assessed pre- and post-treatment (Snaith–Hamilton Pleasure Scale).

**Results:**

Results revealed a significant increase in music-evoked emotion following treatment with psilocybin that correlated with post-treatment reductions in anhedonia. A post-treatment reduction in NAc FC with areas resembling the default mode network was observed during music listening (vs no music).

**Conclusion:**

These results are consistent with current thinking on the role of psychedelics in enhancing music-evoked pleasure and provide some new insight into correlative brain mechanisms.

## Introduction

Music has been part of human cultures for thousands of years (Conard et al., 2009; Nettl, 1956), and modern neuroimaging studies indicate that music listening activates mesolimbic and limbic circuits that underpin the processing of reward and emotion (Blood and Zatorre, 2001; Koelsch et al., 2006; Menon and Levitin, 2005; Mitterschiffthaler et al., 2007). In particular, the ventral striatum (VS) responds to various pleasant and rewarding stimuli (Knutson and Cooper, 2005) and its activation has been associated with the magnitude of emotional arousal to music (Blood and Zatorre, 2001; Trost et al., 2012) and physiologically measurable music-induced ‘chills’ (Blood and Zatorre, 2001). Importantly, the VS has tight structural and functional connections with other regions that subserve reward and emotional processing such as the amygdala, orbitofrontal cortex and insula, lending suggestion as to why music can be both pleasurable and elicit potent and complex emotional responses (Blood and Zatorre, 2001; Krumhansl, 1997).

Listening to music has been shown to engage areas of the brain associated with reward, emotion and memory processing (Barrett and Janata, 2016; Blood and Zatorre, 2001; Salimpoor et al., 2013). Ventral striatal activity is thought to be largely implicated in musical reward (Blood and Zatorre, 2001; Koelsch et al., 2006; Menon and Levitin, 2005; Mueller et al., 2015; Salimpoor et al., 2013), and in conditions where reward system functioning is abnormal, the ability of music to engage the reward circuitry is also affected. Previous studies have successfully used music to investigate anhedonia – that is, the inability to experience pleasure in daily activities (Keller et al., 2013), both hallmarks of depression (Keedwell et al., 2005; Munkler et al., 2015). In fact, trait anhedonia has been associated with reduced music-evoked pleasure, and this was shown to be a result of altered VS functional connectivity (FC) (Jenkins et al., 2018). It has also been demonstrated in healthy individuals that trait anhedonia correlates negatively with pleasantness ratings of music stimuli and activation of key structures involved in reward processing, including the nucleus accumbens (NAc) (Keller et al., 2013).

Early psychedelic therapists took advantage of these properties of music to facilitate emotional release and peak experiences (Bonny, 1972). Psychedelic therapy is a method by which a patient is administered a classic serotonergic psychedelic – for example, psilocybin – with psychological support and is encouraged to direct their attention inward while listening to a playlist of music, often resulting in profound emotionality and spontaneous psychological insight (Carhart-Harris et al., 2014). Psilocybin, like all classic psychedelics, elicits its principal effects through agonism at the serotonin (5-HT) 2a receptor (Nichols, 2004), widely expressed in the cortex (Varnas et al., 2004), to markedly alter consciousness, including changes in perception and emotionality (Carhart-harris et al., 2016; Hasler et al., 2004).

Psychedelic therapy is presently gaining traction as a mental health intervention in diverse populations (Nutt and Carhart-Harris, 2020), including in samples of individuals with unipolar depression (Carhart-Harris et al., 2016; Davis et al., 2020; Osorio et al., 2015). In a therapeutic context, psychedelics appear to work in concert with music to enhance emotionality, personal meaning and mental imagery (Kaelen et al., 2017, 2018; Preller et al., 2017). The interaction between psychedelics and music is believed to be a key component of psychedelics’ therapeutic action (Kaelen et al., 2018).

The default mode network (DMN) is a network of functionally and structurally connected brain regions that exhibits especially high metabolic activity, and relative decreases in activity when individuals engage in goal-directed (i.e. cognitively demanding) cognition and action (Raichle et al., 2001). The DMN has been associated with a broad range of mental phenomena; these could be summarized as spontaneous and internally generated thought (Smallwood et al., 2016). The DMN may be relevant to the therapeutic action of psychedelics as its functioning has been found to altered within the psychedelic state (Carhart-Harris et al., 2012; Palhano-Fontes et al., 2015) as well as after psychedelic therapy for depression (Carhart-Harris et al., 2017). Abnormal DMN functioning has been linked to depression and anxiety, as well as negative self-referential thoughts (Berman et al., 2011; Dos Santos et al., 2016; Greicius et al., 2007; Sheline et al., 2009), rumination and anhedonia (Bluhm et al., 2009; Greicius et al., 2007; Jenkins et al., 2018; Sheline et al., 2009).

There is a lack of neuroscientific research pertinent to the hypothesized interaction between music listening and psychedelic therapy. The present study sought to address this knowledge gap by assessing whether patients with depression show altered VS connectivity during music listening after versus before treatment with psilocybin. This was part of a larger project assessing the feasibility, safety and efficacy of psilocybin in patients with unipolar treatment-resistant depression (Carhart-Harris et al., 2016), where patients underwent functional magnetic resonance imaging (fMRI) before and after treatment.

## Experimental procedures

### Approvals

This study was part of an open-label feasibility trial investigating the safety and efficacy of psilocybin for treatment-resistant depression (Carhart-Harris et al., 2016). The study was sponsored and approved by the Joint Research and Compliance Office of Imperial College London and complied with the International Committee on Harmonisation Good Clinical Practice (GCP) guidelines, the National Health Service Research Governance Framework, and with the ethical standards of the revised declaration of Helsinki (2000). The study was also approved by the National Research Ethics Service ethics committee (West London) and the Medicines and Healthcare products Regulatory Agency. A Home Office license was obtained to conduct research with schedule 1 drugs. Consistent with GCP, all patients provided written informed consent.

### Participants

Nineteen participants (13 males and 6 females; mean age ± SD = 43.1 ± 10.5; range = 27–64) completed this study. Screening for physical and mental health took place prior to study inclusion. Physical health assessments comprised of an electrocardiogram, blood tests, urine tests for recent drug use and pregnancy and a comprehensive medical history review. Mental health assessments comprised of a psychiatric interview, clinician assessments of depression severity and patient-rated scales of depressive symptoms. Key inclusion criteria for the study were diagnosis of moderate to severe depression, as determined by a score of 17 or higher on the 21-item Hamilton Depression Rating Scale, with absence of improvements despite at least two different pharmacological antidepressant treatments for a minimum of 6 weeks within the current depressive episode (Sackeim, 2001). Key exclusion criteria were as follows: current or previously diagnosed psychotic disorder, diagnoses of psychotic disorders in immediate family members, history of suicide attempts that required hospitalization, history of mania, having a blood or needle phobia, pregnancy and current substance or alcohol use disorders. Participants were asked to stop their medication for the trial, to avoid suspected attenuation of psilocybin’s effects (Bonson et al., 1996). This was done in a tapered manner under careful supervision from the study psychiatrist. Washout occurred over at least 2 weeks prior to study entry.

All participants provided written informed consent. For details on patients’ baseline and demographic characteristics and clinical outcomes, refer Carhart-Harris et al.’s (2016) study and Supplementary Material.

### Study design

Treatment consisted of two separate days on which psilocybin was administered, separated by 1 week. Prior to the first therapy session, preparation was provided via discussion of (a) the patient’s personal history with depression, (b) psilocybin’s psychological effects and (c) what patients could expect for the dosing session. Patients received a low oral dose (10 mg) of psilocybin on the first therapy session and a high dose (25 mg) on the second therapy session, after which they were invited to relax in a reclined position in a hospital room with low lighting that had been decorated for the session (Johnson et al., 2008). Patients listened to a predefined playlist of music (Kaelen et al., 2018), wore eyeshades and were encouraged to focus their attention inward.

Patients were scanned on two separate occasions, 1 week prior to the first therapy session and 1 day following the last therapy session. Upon arrival on each scanning day, patients completed the Snaith–Hamilton Pleasure Scale (SHAPS), a measure of hedonic capacity (Snaith et al., 1995). On each scanning day, two 8-min scans were acquired: the first scan was performed without listening to music and the second while listening to music. The order of the scans was not randomized between patients. Music was presented through MRI-compatible headphones (MR Confon, Magdeburg, Germany), and all scanning occurred under eyes-closed conditions. Immediately following each scan, patients were asked to open their eyes and respond to a question presented on a screen, assessing their experience of pleasure during the prior scanning. The question was formulated ‘How pleasurable was the previous scan?’, and ratings were provided via a response box and on a visual analogue scale, from 0 (‘not at all’) to 10 (‘extremely pleasurable’).

Following each scan, patients completed the 21-item Geneva Emotional Music Scale (GEMS), a validated metric that measures music-evoked emotions (Zentner et al., 2008). Patients were explicitly instructed to rate the GEMS in terms of how they personally felt *in response to* the music, not in relation to what they thought the music was intended to communicate to them, or how they felt in general. The GEMS-21 consists of 21 items that are subsequently calculated into nine distinct factors of music-evoked emotion: wonder (filled with wonder, dazzled, moved), transcendence (fascinated, overwhelmed, feelings of transcendence and spirituality), power (strong, triumphant, energetic), tenderness (tender, affectionate, in love), nostalgia (nostalgic, dreamy, melancholic), peacefulness (serene, calm, soothed), joyful activation (joyful, amused, bouncy), sadness (sad, sorrowful) and tension (tense, agitated, nervous). Each item was scored from 0 to 4: 0 = ‘not at all’, 1 = ‘somewhat’, 2 = ‘moderately’, 3 = ‘quite a lot’ and 4 ‘very much’.

### Stimuli

Two stimuli were compiled, using music tracks by composer Carlos Cipa. Stimulus A included the tracks ‘lost and delirious’ and ‘lie with me’; stimulus B included ‘wide and moving’ and ‘the dream’. The stimuli were balanced for their emotional potency by ratings from an independent sample prior to the study (using the GEMS and ratings for general liking and familiarity). Each stimulus was listened to on the first day and the other on the second, with the order of the playlists counterbalanced across participants. To minimize interference of fMRI scanning noise with the music listening, volume maximization and broadband compression were carried out using Ableton live 9 software.

### FMRI data acquisition and pre-processing

fMRI scans were performed on a 3T Siemens TrioTim System at Imanova (London, UK). An anatomical scan, with an axial orientation, field of view (FOV) = 256 × 256 mm^2^, and a 1 mm isotropic voxel resolution (repetition time (TR)/echo time (TE) = 2300/2.98 ms; inversion time = 900 ms; flip angle = 9°), was first acquired for registration and segmentation. For the functional scans, images were obtained using a T2*-weighted gradient echo planar sequence. Thirty-six slices of 3 mm thickness were acquired in an interleaved fashion with the following parameters: TR/TE = 2000/31 ms; FOV = 192 × 192 mm^2^; voxel dimensions of 3 mm × 3 mm × 3 mm; parallel acceleration factor = 2; flip angle = 80°; bandwith = 2298 Hz/pixel; GRAPPA acceleration = 2 and number of volumes = 240, 8 min.

### blood oxygenation level dependent (BOLD) pre-processing

Four different but complementary imaging software packages were used to analyse the fMRI data. Specifically, FMRIB Software Library (FSL) (Smith et al., 2004), Analysis of Functional NeuroImages (AFNI) (Cox, 1996), Freesurfer (Dale et al., 1999) and Advanced Normalization Tools (ANTS) were used. Fifteen subjects were included in this analysis: one subject was discarded from the analysis due to an injury in parietal cortex and three subjects were discarded due to high levels of head movement. Principally, motion was measured using fram-wide displacement (FD) (Power et al., 2014). The criterion for exclusion was subjects with >20% scrubbed volumes with a scrubbing threshold of FD = 0.5. For the 15 subjects that were used in the analysis, two-tailed paired-sample *t*-tests were conducted, revealing no significant difference in the mean FD before and after treatment (meanFD_before_ = 0.180 ± 0.089, meanFD_after_ = 0.158 ± 0.084, *p* = 0.23). The mean percentage of scrubbed volumes for before and after treatment was 4.6 ± 5 and 3.5 ± 5.2%, respectively (*p* = 0.56). The maximum of scrubbed volumes for before and after treatment was 17.3 and 17.7%, respectively. The following pre-processing stages were performed: (1) removal of the first three volumes; (2) de-spiking (3dDespike, AFNI); (3) slice time correction (3dThift, AFNI); (4) motion correction (3dvolreg, AFNI) by registering each volume to a single volume, which was the volume most similar, in the least squares sense, to all others (in-house code); (5) brain extraction (Brain Extraction Tool [BET], FSL); (6) rigid body registration to anatomical scans (Boundary-Based Registration [BBR], FSL); (7) non-linear registration to 2 mm Montreal Neurological Institute (MNI) brain (symmetric normalization, ANTS); (8) scrubbing (Power et al., 2012) using an FD threshold of 0.5, scrubbed volumes were replaced with the mean of the surrounding volumes; (9) spatial smoothing full-width at half-maximum (FWHM) of 6 mm (3dBlurInMask), AFNI); (10) band-pass filtering between 0.01 to 0.08 Hz (3dFourier, AFNI); (11) linear and quadratic de-trending (3dDetrend, AFNI); and (12) regressing out nine nuisance regressors (all nuisance regressors were band-pass filtered with the same band-pass filter as above): out of these, six were motion related (three translations and three rotations) and three were anatomically related (not smoothed). Specifically, the anatomical nuisance regressors were as follows: (1) ventricles (Freesurfer, eroded in 2 mm space), (2) draining veins (FSL’s CSF minus Freesurfer’s ventricles, eroded in 1 mm space) and (3) local white matter (WM) (FSL’s WM minus Freesurfer’s subcortical grey matter structures, eroded in 2 mm space). Regarding local WM regression, AFNI’s 3dLocalstat was used to calculate the mean local WM time-series for each voxel, using a 25-mm radius sphere centred on each voxel (Jo et al., 2010).

Two parallel datasets were pre-processed using the above method: the first was in MNI space as conventional practice for voxel-wise group analysis and the second in native space in order to extract a time course for the NAc seed. For the latter dataset, all pre-processing stages except 7 and 9 were performed.

### Seed-based FC

For this analysis, the VS, and specifically the NAc, was chosen as the region of interest (ROI). The NAc seed was drawn automatically in native space using Freesurfer parcellation; this was done for each patient individually. Mean time-series were derived for the seed for each scan in an unsmoothed native space. Whole-brain voxel-wise FC analyses were performed using FSL’s FEAT for each subject (generalized linear model (GLM)). Pre-whitening (FILM) was applied. A higher level analysis was performed to compare pre-treatment and post-treatment conditions using a mixed-effects GLM at the group level (FMRIB’s Local Analysis of Mixed Effects [FLAME] 1 + 2). *Z* statistical parametric maps were thresholded using clusters determined by *z* > 2.3 and a whole-brain significance threshold of *p* < 0.05. MRIcron was used to display the results.

### Data analysis

All statistical tests were performed in Statistical Package for the Social Sciences for Mac, Version 23.0. Graphpad Prism 8 for Mac was used to illustrate the results. Changes in NAc FC were assessed by performing a two-way repeated measures analysis of variance (ANOVA) (using mean *Z*-stat scores of each participant within a mask of the significant FC group result) to test for differences between music- and no music scans (a music-effect), before and after treatment (a treatment-effect), and for an interaction-effect between music- and treatment-effect corresponding to in-scanner pleasure ratings (i.e. for the question ‘How pleasurable was the previous scan?’). Following the ANOVA, paired two-tailed *t-*test was performed to test for significant differences between conditions for all in-scanner ratings. Multiple *t*-tests were conducted to assess differences between conditions during the music scan (after treatment – before treatment) for the nine factors calculated by the GEMS-21. False discovery rate (FDR) control was used to correct for multiple comparisons (Benjamini and Hochberg, 1995).

Both one- and two-tailed Pearson’s r correlation analyses were performed to explore the relationship between changes in mean in-scanner pleasure ratings during music listening after treatment with psilocybin (baseline vs 1 day after the 25 mg dose) and corresponding changes in FC of the NAc with the areas resembling the DMN using mean *Z*-stat scores for the ROI of each participant ((music – no music) – (after treatment – before treatment)), as well as changes in anhedonia scores between baseline and the three different time points (i.e. 1 day, 1 week or 3 months after treatment). Standard criteria for outlier removal were followed (Robust regression and Outlier removal [ROUT]; Q-1%) for all analyses.

## Results

### The effects of psychedelic therapy on music-evoked pleasure

**Figure 1.**
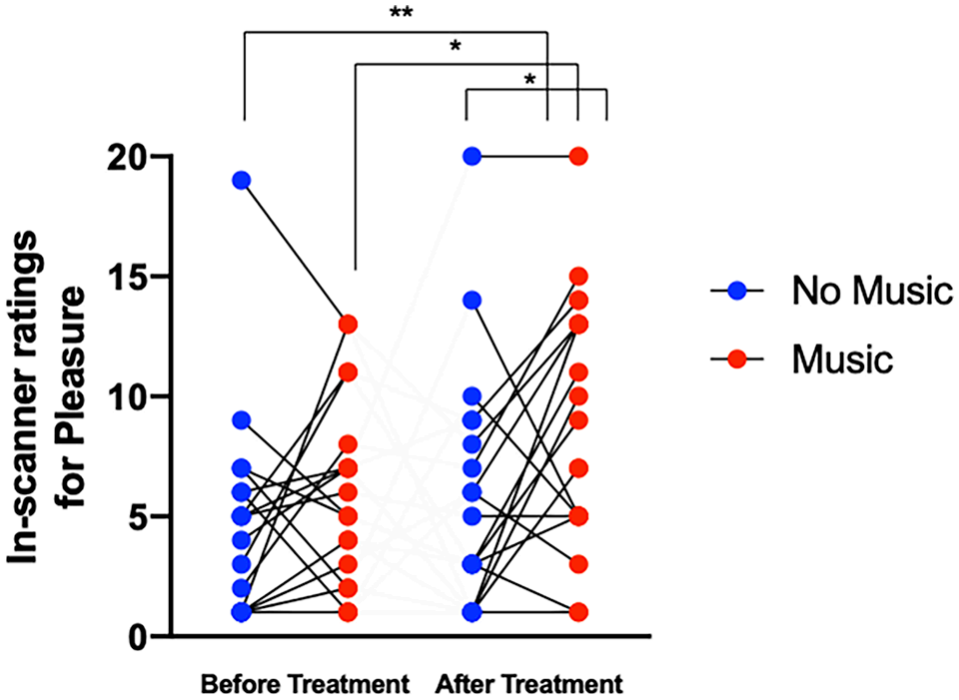
Individual values for in-scanner ratings of pleasure during no music scans (blue) and music scans (red), before treatment (left) and after (right) treatment. A two-way repeated measures ANOVA music effect (*F*(1,18) = 5.74, *p* = 0.0277), but no treatment effect (*F*(1,18) = 3.857, *p* = 0.0652) or interaction effect (*F*(1,18) = 1.846, *p* = 0.191). Follow-up paired *t*-tests demonstrated a significant increase in ratings of pleasure felt when listening to music post treatment relative to before (**p = 0.0083, *t* = 2.963, df = 18), increased ratings of pleasure between no music and music scans post-treatment (*p = 0.0195, *t* = 2.565, df = 18), as well as increased ratings of pleasure between no music before treatment and music- after treatment (**p = 0.0081, *t* = 2.973, df = 18). ANOVA: analysis of variance.

A two-way repeated measures ANOVA on the in-scanner pleasure ratings (i.e. in-scanner ratings for the question ‘How pleasurable was the previous scan), revealed a music effect (*F* = 5.74, df = 18, *p* = 0.0277), but no treatment (*F* = 3.857, df = 18, *p* = 0.0652) or interaction-effect between treatment and music (*F* = 1.846, df = 18, *p* = 0.191). Follow-up paired *t*-tests comparing pleasure ratings for the music scans revealed significantly greater pleasure scores post-treatment (9.00 ± 5.25, *t* = 2.963, df = 18, *p* = 0.0083) than pre-treatment (5.84 ± 3.95). Paired *t*-tests also revealed a significant increase in ratings for pleasure post-treatment, between no music (5.84 ± 5.06) and music scans (9.00 ± 5.25, *t* = 2.565, df = 18 *p* = 0.0195), as well as increased ratings of pleasure between no music before treatment (4.47 ± 4.36) and music after treatment (9.00 ± 5.25, *t* = 2.973, df = 18, *p* = 0.0081) (Figure 1).

### The effects of psychedelic therapy on different types of music-evoked emotion

**Figure 2.**
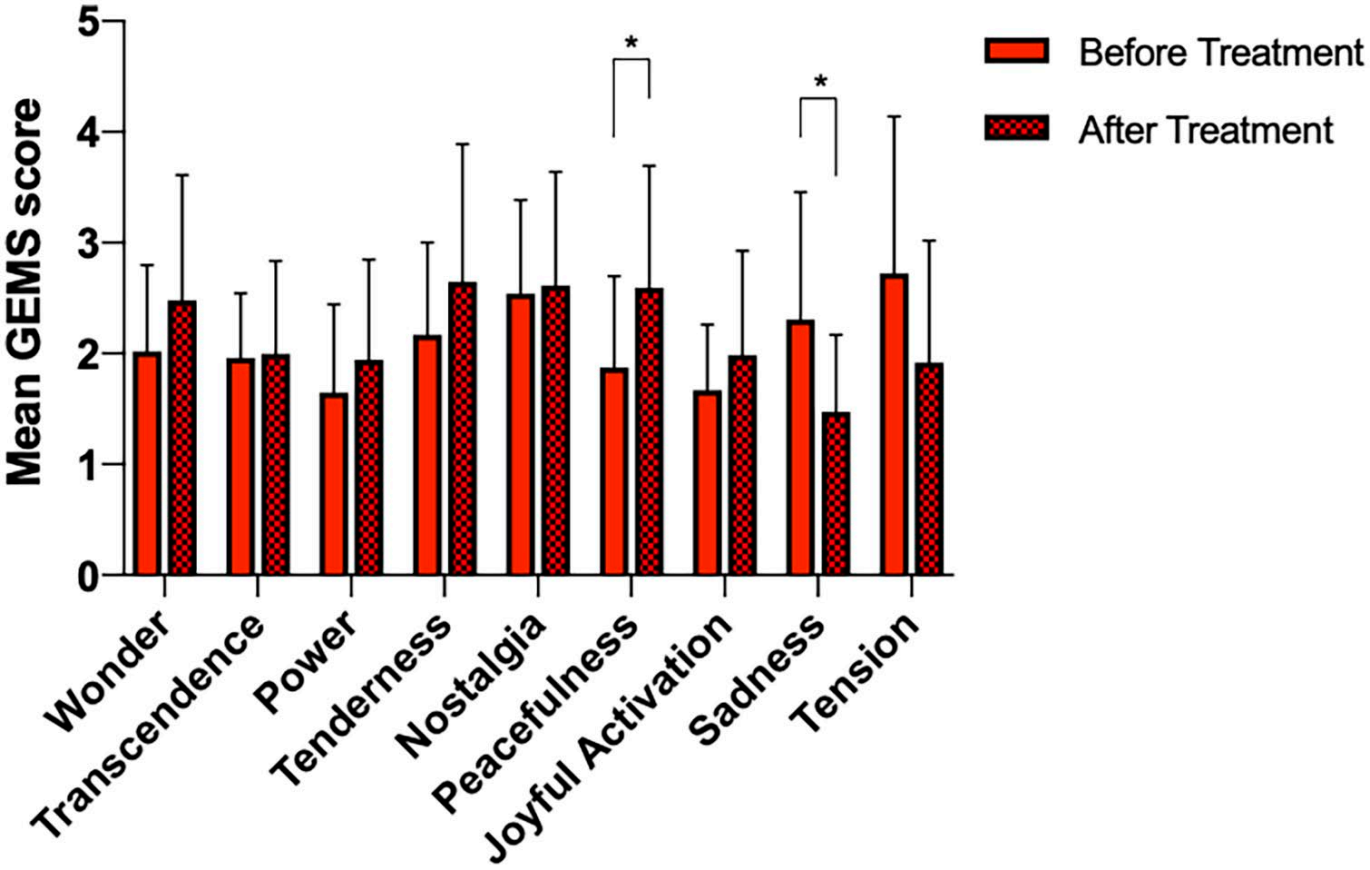
Mean values plus standard deviations for the GEMS-9 scores following the music scans: before treatment (red) and after treatment (checkered red). Multiple paired *t*-tests demonstrated significantly lower scores following treatment for the item Sadness (*p* = 0.0123, *t* = 2.626, df = 34) and a significant increase for the item Peacefulness (*p* = 0.0328, *t* = 2.226, df = 34). Reported *p*-values are FDR adjusted, with significance threshold *p* = 0.05 after correction. * *p* <0.05.

Multiple paired *t*-tests revealed a significant decrease in the GEMS factor music-evoked ‘Sadness’ post-treatment (before: 2.34 ± 1.13, after: 1.47 ± 0.68; *t* = 2.626, df = 34, *p* = 0.0123). A significant increase was also detected in the GEMS factor music-evoked ‘Peacefulness’ (before: 1.88 ± 0.80, after: 2.54 ± 1.09; *t* = 2.226, df = 34 *p* = 0.0328) (Figure 2). FDR correction for multiple comparisons was applied, with significance threshold *p* = 0.05 after correction.

### The effects of psychedelic therapy on VS FC

**Figure 3.**
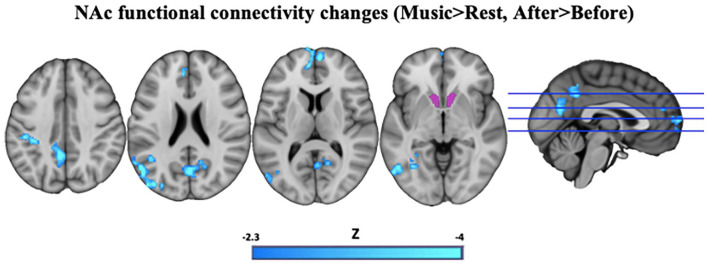
Seed-based FC analysis with the bilateral NAc seed (purple, for illustration purposes only), showing significantly less FC post-treatment (blue) versus pre-treatment during music listening versus no music listening. Decreased NAc FC is evident within areas resembling the DMN. Cluster correction was applied to all images with a threshold of *p* < 0.05, *Z* >−2.3. Left side represents left hemisphere. FC: functional connectivity; DMN: default mode network; NAc: nucleus accumbens.

Seed-based FC analyses (mixed-effects GLM) revealed decreased FC between the bilateral NAc seed and regions resembling the DMN post-treatment compared to pre-treatment during music listening versus no music listening (Figure 3).

**Figure 4.**
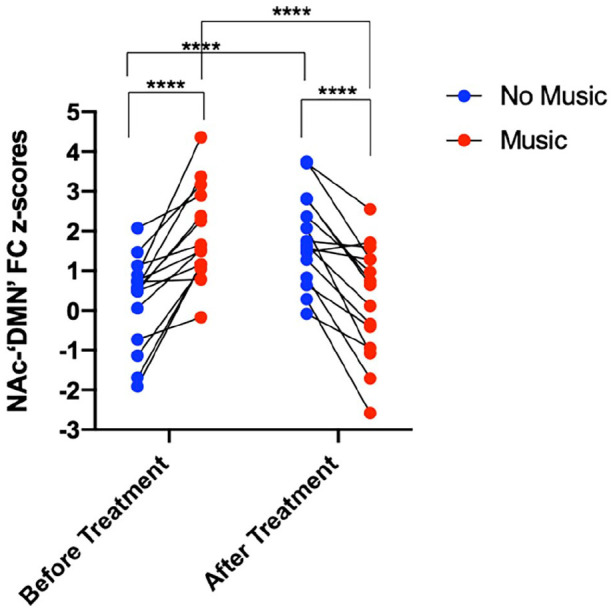
NAc-‘DMN’ FC values (*z*-scores displayed on *y*-axis) of each individual participant (each dot representing one participant, *n* = 15) for each scan (blue = no music, red = music), for each condition (‘before treatment’; left) and ‘after treatment’; right). A two-way repeated measures ANOVA revealed a significant interaction between treatment and music (*F*(1,14) = 115.7, *p* <0.0001). Follow-up paired *t*-tests revealed significantly greater NAc-‘DMN’ FC for the music versus no music scans before psilocybin treatment (*p* <0.0001, *t* = 5.956, df = 14). After treatment, there was a significant decrease in NAc-‘DMN’ FC between the no music versus music scans (*p* <0.0001, *t* = 5.861, df = 14). Furthermore, significantly greater NAc-‘DMN’ FC was evident when comparing the music scans after versus before treatment (*p* <0.0001, *t* = 6.135, df = 14) as well as the no music scans after versus before treatment (*p* <0.0001, *t* = 4.918, df = 14). **** *p* <0.0001. FC: functional connectivity; DMN: default mode network; NAc: nucleus accumbens; ANOVA: analysis of variance.

The two-way repeated measures ANOVA on FC in NAc with the areas resembling the DMN (using mean *Z*-stat scores of each participant within a mask of the significant FC group result) in different conditions ((music – no music) – (after treatment – before treatment)) revealed a significant interaction between treatment and music (*F*(1,14) = 115.7, *p* < 0.0001). Assessing FC separately for each time point (before and after treatment) and scan (no music and music) via follow-up paired *t*-tests showed significantly greater coupling between the NAc and regions resembling the DMN between the music and no music scans prior to treatment (*p* < 0.0001, *n* = 15, df = 14). After treatment, there was significantly less NAc-‘DMN’ coupling in the no music scan (*p* < 0.0001, *n* = 15, df = 14) and the music scan (*p* < 0.0001, *n* = 15, df = 14). When comparing the scans after versus before treatment, there was significantly greater NAc-‘DMN’ coupling in both the music (*p* < 0.0001, *n* = 15, df = 14) and the no music scans (*p* < 0.0001, *n* = 15, df = 14) (Figure 4).

### Correlation analyses

To assess a potential relationship between changes in in-scanner pleasure ratings and changes in NAc FC following psilocybin treatment, during music, a Pearson correlation test was conducted, revealing no statistically significant relationship between changes in mean in-scanner pleasure ratings ((music – no music) – (after treatment – before treatment)) and changes in NAc FC values ((music – no music) – (after treatment – before treatment)) (*r* = −0.0135, *n* = 15, *p* = 0.9619) (not shown).

### Test–retest reliability

In order to assess the reliability of the pleasure ratings data measured over time, Pearson correlation coefficients were calculated for the music (after treatment – before treatment) group as well as no music (after treatment – before treatment) group. Results revealed fair test–retest reliability in the music group (*r* = 0.5201, *n* = 19, *p = 0.0112), but not the no music group (*r* = −0.01656, *n* = 19, *p* = 0.4732) (not shown). The same was done for the NAc-DMN coupling data, for the music (after treatment – before treatment) group as well as no music (after treatment – before treatment) group, revealing good test–retest reliability in the music group (*r* = 0.7007, *n* = 15, **p = 0.0018), but not the no music group (*r* = 0.4364, *n* = 15, *p* = 0.0519) (not shown).

### Anhedonia results

**Figure 5.**
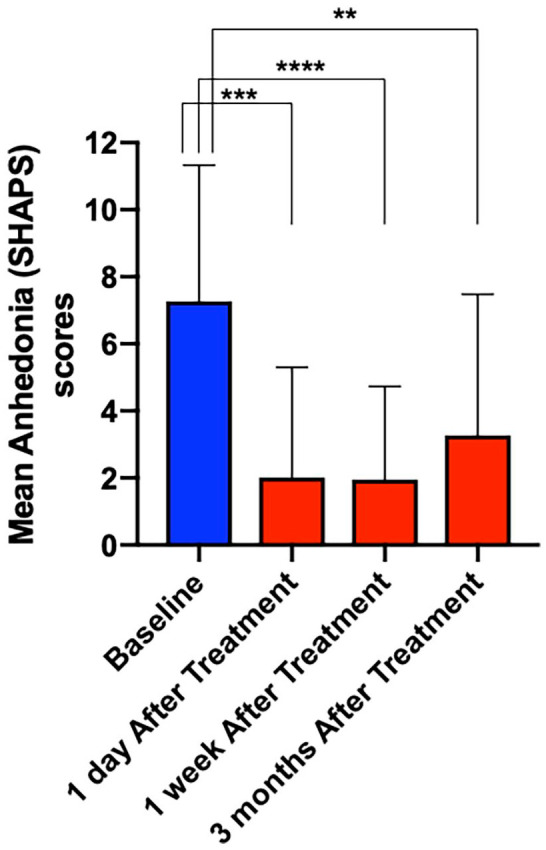
Mean values plus standard deviations for anhedonia at baseline (blue) and 1 day, 1 week and 3 months following treatment. A one-way repeated measures ANOVA analysis revealed a significant difference between the groups (*F*(3,72) = 4.096, *p* < 0.0001). Follow-up paired *t*-tests demonstrated a significant decrease in anhedonia scores post treatment relative to baseline (1 day after *p* = 0.0002, *t* = 4.696, df = 18; 1 week after *p* < 0.0001, *t* = 5.362, df = 18; 3 months after *p* = 0.0026, *t* = 3.487, df = 18), all of which survive Bonferroni correction for multiple comparisons. ** *p* <0.01, *** *p* <0.001 and **** *p* <0.0001. ANOVA: analysis of variance.

A one-way ANOVA revealed a significant difference in anhedonia scores taken at different time points (baseline and 1 day, 1 week, and 3 months following psilocybin treatment) as measured by the SHAPS (*F*(3,72) = 4.096, *p* < 0.0001) (Figure 5). Follow-up paired *t*-tests revealed statistically significant decreases in anhedonia scores from baseline to 1 day (*p* = 0.0002, *t* = 4.696, df = 18), 1 week (*p* < 0.0001, *t* = 5.362, df = 18) and 3 months (*p* = 0.0026, *t* = 3.487, df = 18) following psilocybin treatment – all of which survived Bonferroni correction for multiple comparisons (i.e. revised *p* *=* 0.05/3 = 0.016).

**Figure 6.**
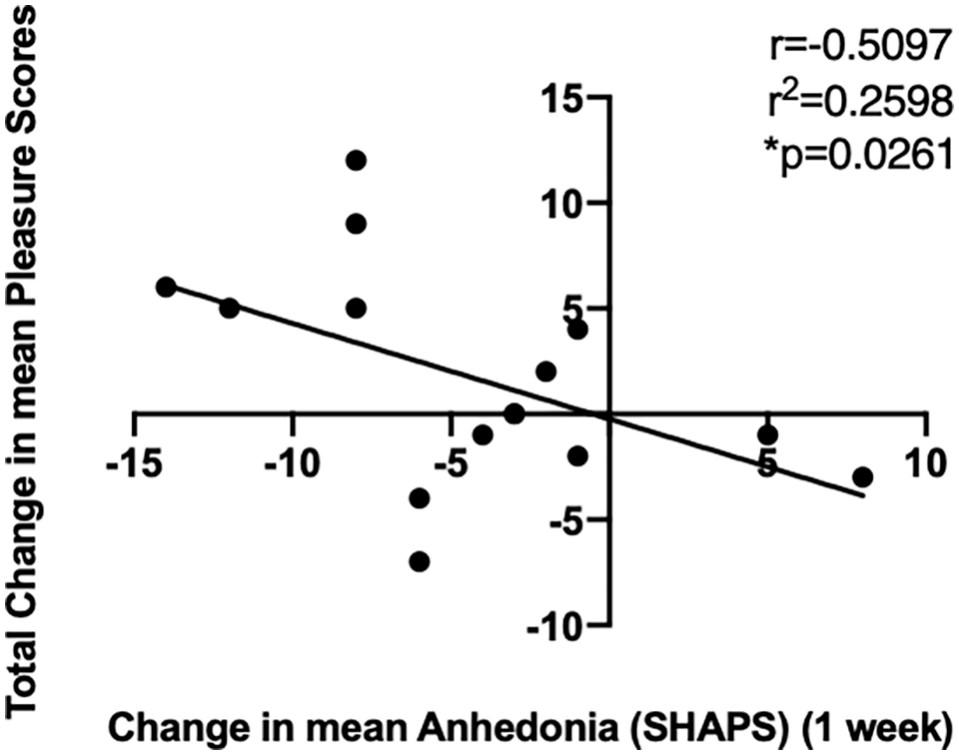
Correlation analyses revealed a negative relationship between changes in pleasure ratings ((music – no music) – (after treatment – before treatment)) and changes in anhedonia scores 1 week following treatment (compared to baseline), which reached statistical significance (*r* = −0.5264, *n* = 15, *p* = 0.0438). * *p* <0.05.

In order to assess whether there was a relationship between music-induced pleasure ratings following psilocybin treatment and corresponding changes in anhedonia scores, two-tailed Pearson correlation analyses were conducted by calculating the total changes in anhedonia scores between baseline and the three different time points (i.e. 1 day, 1 week or 3 months after treatment) and total changes in pleasure scores ((music – no music) – (after treatment – before treatment)). Results revealed a statistically significant negative correlation between changes in pleasure ratings and changes in anhedonia scores 1 week following treatment (*r* = −0.52, *p* = 0.0438) (Figure 6). In other words, the higher the music-induced pleasure scores following treatment with psilocybin, the lower the anhedonia scores 1 week following treatment.

Finally, two-tailed Pearson correlation tests were conducted to assess a potential relationship between changes in anhedonia scores and changes in NAc FC following psilocybin treatment, during music. No relationships were found between changes in mean anhedonia scores (baseline vs 1 day, 1 week and 3 months following psilocybin treatment) and changes in NAc-‘DMN’ FC values ((music – no music) – (after treatment – before treatment)).

## Discussion

The present investigation sought to examine whether psilocybin therapy for treatment-resistant depression could elicit post-treatment changes in music-evoked emotion, as well as changes in NAc (or ‘ventral striatal’) FC. As predicted, post hoc linear contrasts revealed significantly greater felt pleasure to music post-treatment versus pre-treatment, although there was no interaction between condition (music vs no music) and time (before vs after treatment). A significant decrease in music-evoked sadness and a significant increase in music-evoked peacefulness were seen post-treatment with psilocybin. A high-level interaction analysis comparing music (vs no music) pre- vs. post-psilocybin treatment revealed a contrasting effect of music (vs no music) on NAc FC post-treatment versus pre-treatment with psilocybin. Specifically, a post-treatment decrease in NAc FC with regions resembling the DMN pre-treatment was observed, which contrasted with an increase in NAc-‘DMN’ coupling during music listening pre-treatment. These changes in NAc FC did not correlate with changes in in-scanner music-evoked pleasure ratings post-treatment with music. Finally, sustained decreases in anhedonia scores were observed post-treatment which correlated with post-treatment increases in music-evoked pleasure. These results validate music-evoked pleasure as a paradigm for assessing anhedonia severity in depressed patients, and the sensitivity of both to improvement post-psilocybin therapy. The results also highlight candidate neural mechanisms pertinent to altered processing of pleasurable stimuli post psilocybin therapy. However, further work is needed to elucidate the functional relevance of the observed brain changes, as they did not correlate with the relevant psychological measures.

### Music-evoked emotions and anhedonia

Our findings of decreased music-evoked ‘sadness’ and increased music-evoked ‘peacefulness’ are consistent with previous studies illustrating the ability of psychedelics to enhance music-evoked emotion (e.g. ‘wonder’ and ‘transcendence’) (Kaelen et al., 2015, 2018; Maslow, 1971; Richards, 2009) and the notion that the combination of music and psychedelics may contribute to the occurrence of mystical-type or peak experiences, which is predictive of the therapeutic effects of psychedelics (Griffiths et al., 2008; Garcia-Romeu et al., 2014; Kaelen et al., 2015, 2018; MacLean et al., 2011). Importantly, in the context of sustained changes in music-evoked emotion following psilocybin treatment, the observed reductions in anhedonia scores persisted for up to 3 months and, at 1 week post treatment, correlated with in-scanner pleasure ratings after listening to music. Thus, our results reinforce the existence of a relationship between anhedonia and music-evoked pleasure (Keller et al., 2013; Osuch et al., 2009; Young et al., 2016) and imply positive changes in both following psilocybin treatment. Limiting the interpretability of the present study, however, was our inability to detect a relationship between observed changes in NAc functionally connectivity and either felt pleasure or anhedonia (Jenkins et al., 2018; Keller et al., 2013; Osuch et al., 2009).

### Changes in NAc FC following psilocybin treatment

The fMRI results reported here revealed an overall reduction in NAc FC with regions resembling the DMN during music listening post-treatment with psilocybin. Unpacking this high-level interaction (significance thresholds-aside), there was an increase in NAc-‘DMN’ coupling after (vs. before) treatment in the no music scan (treatment effect) and between the no music and music scans *prior* to treatment (music effect); whereas NAc-‘DMN’ coupling was *decreased* after versus before treatment in the music scan (treatment effect) and in the music versus no music scans *after* treatment (music effect).

Although difficult to interpret, this finding tentatively implicates a role for the NAc and high-level cortical regions with which it is functionally coupled (i.e. those resembling the DMN) in music processing and its sensitivity to psilocybin therapy. Although speculative, it may be that top-down cortical control of the limbic regions such as the NAc is reduced post psilocybin therapy, facilitating music-evoked emotionality post-treatment (Roseman et al., 2018). Such a model would be consistent with previous findings of ours from the same study but a separate paradigm where decreased ventromedial prefrontal cortex (vmPFC) FC with the (right) amygdala was observed during fearful face processing post psilocybin therapy for treatment-resistant depression (Mertens et al., 2020). In fact, the amygdala has previously been implicated in the pathophysiology of depression (Drevets et al., 1992), being one of the areas implicated in processing, detecting and expressing emotions, including music (Koelsch, 2014), and so this might also be an area of interest in future investigations. The present results could also be compared with previous findings in depression, where, for example, greater DMN resting-state FC was found in depressed individuals compared with healthy controls (Greicius et al., 2007; Hamilton et al., 2011; Li et al., 2013). Similarly, Hwang et al. (2016) found significantly greater DMN-NAc FC in mildly depressed individuals, compared with healthy controls (Hwang et al., 2016). These findings could support the inference that the DMN-NAc circuit is overactive in depression, possibly suppressing NAc-mediated hedonic responses and thus accounting for the phenomenon of anhedonia that is characteristic of the disorder (Keller et al., 2013). The here-observed diminishment of music-induced coupling of the NAc-‘DMN’ circuit post psilocybin treatment may reflect a correction, or normalization, of this overactive inhibitory control, thus accounting for a post-treatment recovery of the normal hedonic response. However, this inference is speculative and was not supported by subsequent correlational analyses. Thus, further scrutiny of the present study’s findings is required before we can offer any reliable inferences about causal mechanisms. Dynamic causal modelling could be employed to test the hypothesis that changes in a presumed top-down DMN-NAc causal influence are involved. It is worth noting, however, that it seems that there is evidence in favour of the reproducibility of this investigation, given relatively high reliability scores in the NAc-DMN coupling data in the Music group (Shou et al., 2022).

Jenkins et al. (2018) showed that high DMN activity differentiated high anhedonia scores in individuals with depression from the relatively lower scores in healthy controls. The authors suggest that failure to downregulate DMN activity may lead to increased negative self-referential processing in depression (Jenkins et al., 2018; Sheline et al., 2009), which could support the notion that hyperactivity of the DMN contributes to increased rumination and greater anhedonia in depression (Bluhm et al., 2009; Greicius et al., 2007; Jenkins et al., 2018).

In separate but related work, Young et al. (2016) examined the relationship between anhedonia and task-modulated posterior (p) vmPFC FC during music listening in depressed individuals and healthy controls and found that anhedonia was differentially associated with pVMPFC connectivity when processing pleasant music stimuli, but not during rest, and not when processing scrambled music (control stimuli). The authors suggest that anhedonia is task or context specific and is associated with a *lack* of engagement between the pVMPFC and reward-related functional circuits when encountering pleasurable stimuli, rather than a constant static deficit in connectivity (Young et al., 2016). In the context of our study, it is important to note that the music chosen was selected for being emotionally moving rather than pleasurable per se – indeed, it was somewhat melancholic in nature, despite the fact that participants reported it as more pleasurable, less sad and more peaceful post-treatment. Future studies might benefit from more dynamic analyses of specific time periods within a piece of music, for example, to isolate periods that are associated with strong emotion, including pleasure.

### Limitations and future directions

The present study is limited by its relatively small sample size and absence of comparison to healthy controls. The study is also limited by its open-label design and the absence of placebo. Moreover, imaging data was acquired only 1 day post-treatment, so it was not possible to assess brain changes over a longer time frame. The absence of a different genre or quality of music as a control condition (e.g., scrambled music) may not have been ideal. Furthermore, levels of pleasure in listening to the preferred musical genre before and after using psilocybin were not evaluated, which might have been useful in further assessing changes in anhedonia. In the future, it might be worthwhile to assess the effect of preferred music (Jenkins et al., 2018), different music types (Osuch et al., 2009), auditory noise (Jenkins et al., 2018), unpleasant music (Koelsch et al., 2006) or even music-induced ‘chills’ (Mori and Iwanaga, 2017). The order of the no music versus music scans was not randomized (although the music playlist was counterbalanced between patients), and thus, order confounds cannot be entirely discounted. Additionally, the baseline (no music) scan did not control for auditory qualities, suggesting the possibility that any effects of the music could be indistinguishable from those associated with general auditory stimulation. Finally, given the tight structural and functional connections between the VS and regions known to be involved in musical reward and emotional processing (e.g. amygdala, orbitofrontal cortex, insula, thalamus), future investigations could focus on exploring those, as well as the NAc.

## Conclusions

In an fMRI setting, here we observed increased music-evoked pleasure post-treatment with psilocybin for depression that correlated with subsequent improvements in anhedonia scores. fMRI analyses revealed reduced NAc FC with regions resembling the DMN post-treatment with psilocybin for depression during the music listening. We tentatively interpret these findings as suggestive of a lifting of an inhibitory influence of the DMN on the NAc post-treatment with psilocybin; however, the results of correlational analyses were not supportive of this interpretation. Further research is therefore needed to better understand how recovery of hedonic responsiveness is encoded in the brain after treatment with psilocybin therapy for depression, although music listening does appear to be an effective means of probing this.

## Supplemental Material

sj-docx-1-jop-10.1177_02698811221125354 – Supplemental material for Changes in music-evoked emotion and ventral striatal functional connectivity after psilocybin therapy for depression Click here for additional data file. Supplemental material, sj-docx-1-jop-10.1177_02698811221125354 for Changes in music-evoked emotion and ventral striatal functional connectivity after psilocybin therapy for depression by Melissa Shukuroglou, Leor Roseman, Matt Wall, David Nutt, Mendel Kaelen and Robin Carhart-Harris in Journal of Psychopharmacology
