# Supplementary material for: Changes in music-evoked emotion and ventral striatal functional connectivity after psilocybin therapy for depression
Source: J Psychopharmacol. 2022 Nov 26;37(1):70–9. doi: 10.1177/02698811221125354 (PMC9834320; doi:10.1177/02698811221125354)
Supplement: sj-docx-1-jop-10.1177_02698811221125354 – Supplemental material for Changes in music-evoked emotion and ventral striatal functional connectivity after psilocybin therapy for depression [file sj-docx-1-jop-10.1177_02698811221125354.docx]

**Supplementary Material**

|  |  |  |  | **Baseline Scores** | | |  |  |  |  |  |
| --- | --- | --- | --- | --- | --- | --- | --- | --- | --- | --- | --- |
| **Age** | **Ethnicity** | **Employment Status** | **Estimated illness duration, years** | **BDI** | **HAM-D** | **STAI** | **Past unsuccessful medications** | **Past psychotherapy** | **Education** | **Weekly alcohol intake, units** | **Previous psilocybin use (time since last use)** |
| 43 | Black Caribbean | Employed | 30 | 36 | 19 | 72 | SSRI (two), SNRI (two), NDRI, NSSRI, MAOI | None | Postgraduate | 1 | 0 |
| 40 | Hispanic | Unemployed | 25 | 33 | 28 | 76 | SSRI (two), SNRI, NDRI, NSSRI, Na+ channel blocker (two), ketamine, TCA | Cognitive narrative therapy | Postgraduate | 0 | 0 |
| 37 | White | Employed | 17 | 22 | 18 | 63 | SSRI (two), SNRI | Cognitive behavioural therapy | Postgraduate | 0 | 0 |
| 30 | White | Studying | 10 | 26 | 18 | 67 | NDRI, NSSRI | Cognitive behavioural therapy | Postgraduate | 0 | One use (6 months) |
| 34 | White | Unemployed | 12 | 38 | 25 | 71 | SSRI (three), TCA | Cognitive behavioural therapy, Mindfulness | Undergraduate | 0 | 0 |
| 57 | White | Unemployed | 29 | 39 | 23 | 78 | SSRI (four), SNRI, SARI | Psychotherapy, unclear what | Undergraduate | 2 | Two uses (45 years) |
| 52 | White | Unemployed | 27 | 33 | 22 | 57 | TCA, SARI | Counselling | Secondary Education | 0 | Three uses (30 years) |
| 37 | White | Employed | 17 | 39 | 17 | 71 | SSRI (two), TCA | Counselling | Undergraduate | 2 | 0 |
| 37 | White | Unemployed | 15 | 32 | 26 | 71 | SSRI (three), SNRI | Counselling, Cognitive Behavioural Therapy | Postgraduate | 6 | 0 |
| 36 | Black Caribbean | Unemployed | 8 | 47 | 28 | 75 | SSRI (two), NSSRI | Counselling | Undergraduate (incomplete) | 18 | Three uses (20 years) |
| 64 | White | Employed | 15 | 24 | 16 | 72 | SSRI (four), SNRI (two), NDRI, MAOI, Na+ channel blocker, SARI, DRI | Psychotherapy - various modalities | PhD | 1 | Three uses (50 years) |
| 45 | White | Employed | 8 | 35 | 17 | 68 | SSRI, TCA | Cognitive behavioural therapy | Undergraduate | 0 | 0 |
| 27 | White | Employed | 7 | 29 | 26 | 55 | SSRI, TCA, SARI, NDRI | Cognitive behavioural therapy | Postgraduate | 8 | 0 |
| 49 | White | Unemployed | 30 | 36 | 29 | 70 | SSRI (four), SNRI, TCA, NDRI | Psychodrama, Jungian therapy, Group therapy | Undergraduate | 0 | One use (25 years) |
| 56 | Black Caribbean | Unemployed | 30 | 44 | 36 | 66 | SSRI, SARI | Cognitive behavioural therapy | Undergraduate | 0 | 0 |
| 42 | White | Unemployed | 22 | 45 | 29 | 69 | SSRI (three), SARI (two), TCA | None | Undergraduate | 0 | 0 |
| 58 | White | Part retired | 10 | 28 | 28 | 61 | SSRI (two), SARI | Psychodynamic, Jungian analysis | Undergraduate | 0 | 0 |
| 62 | White | Retired | 15 | 42 | 24 | 74 | SSRI (two), TCA, pregabalin | Psychodynamic, Jungian analysis | Postgraduate | 15 | 0 |
| 44 | White | Unemployed | 20 | 27 | 28 | 68 | SSRI (three), SARI, SNRI, NA+ channel blocker, TCA, MAOI | Cognitive behavioural therapy, Mindfulness | Undergraduate | 20 | 0 |

BDI=Beck Depression Inventory. HAMD-D=Hamilton Depression Rating scale. STAI-T=State-Trait Anxiety Inventory. SSRI=selective serotonin-reuptake inhibitor. SNRI=serotonin–noradrenaline reuptake inhibitor. NDRI=noradrenaline–dopamine-reuptake inhibitor. NSSRI=noradrenaline and specific serotonin-reuptake inhibitor. MAOI=monoamine oxidase inhibitor. TCA=tricyclic antidepressant. SARI=serotonin antagonist and reuptake inhibitor. DRI=dopamine-reuptake inhibitor. *One medication from each class, unless otherwise stated.

**Table 1.** Baseline and Demographic Characteristics, by patient

**NAc RSFC (No Music, Before)**

**
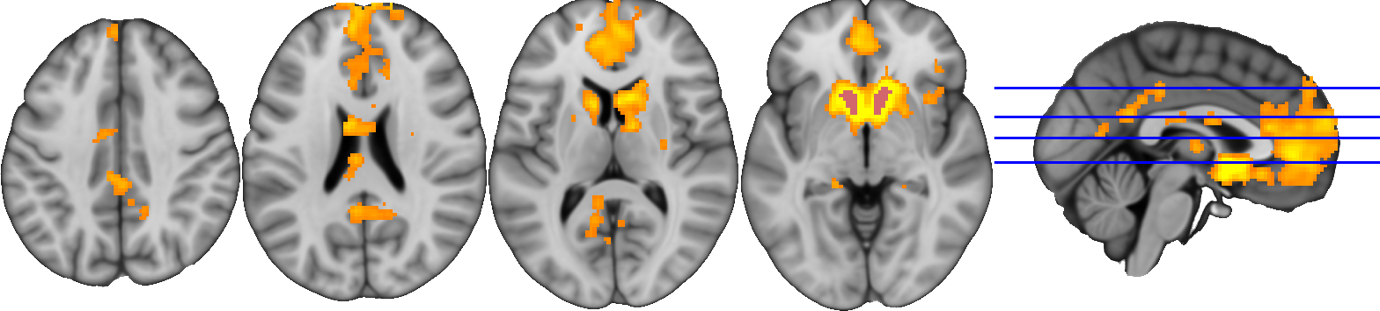
**

**NAc RSFC (Music, Before)**

**
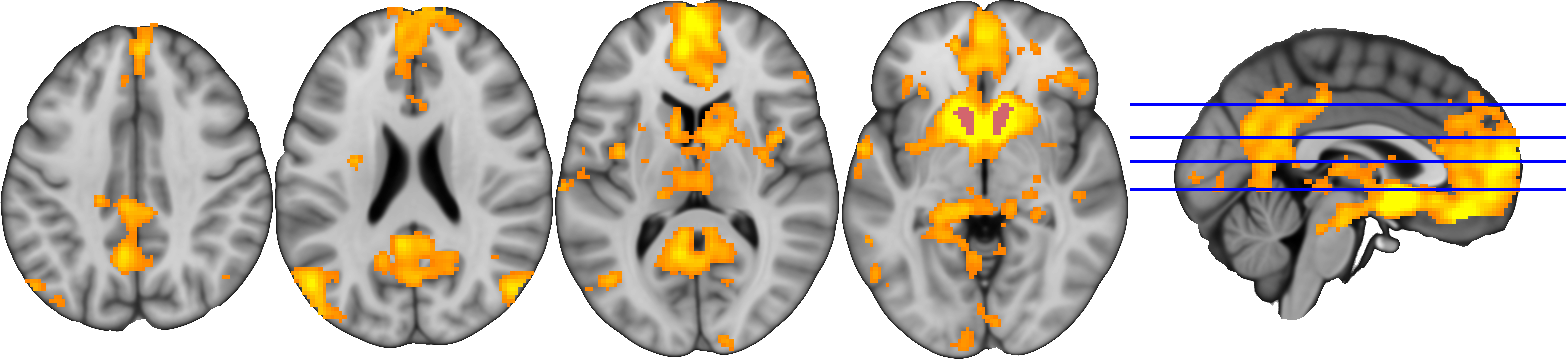
**

**NAc RSFC (No Music, After)**

**
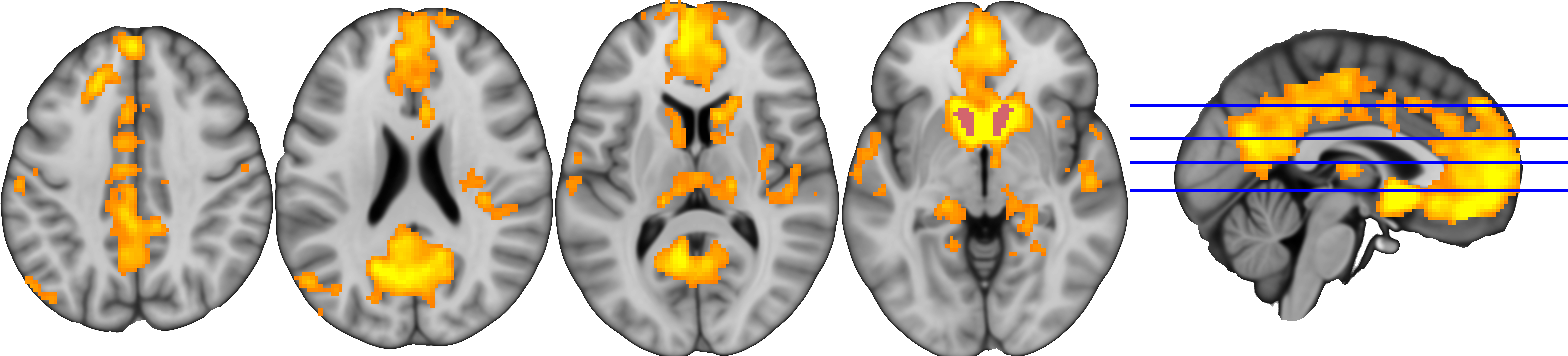
**

**NAc RSFC (Music, After)**

**
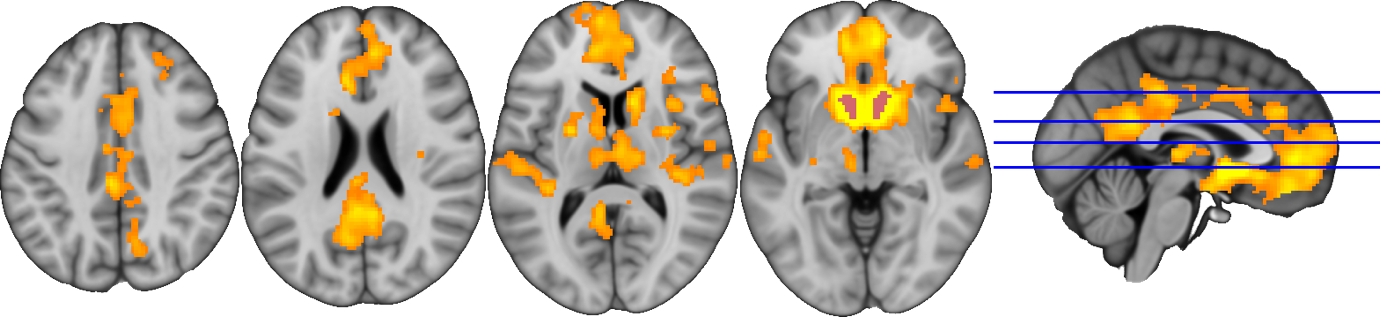
**


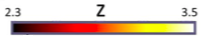


**Figure 1a.** Top two rows = NAc (purple) RSFC before psilocybin treatment, without and with music (hot colours = regions of significantly positive coupling). Bottom two rows = NAc (purple) RSFC after psilocybin treatment, without and with music. Cluster-correction was applied to all images with a threshold of, z>2.3.

**NAc RSFC (Music>Rest, Before)**


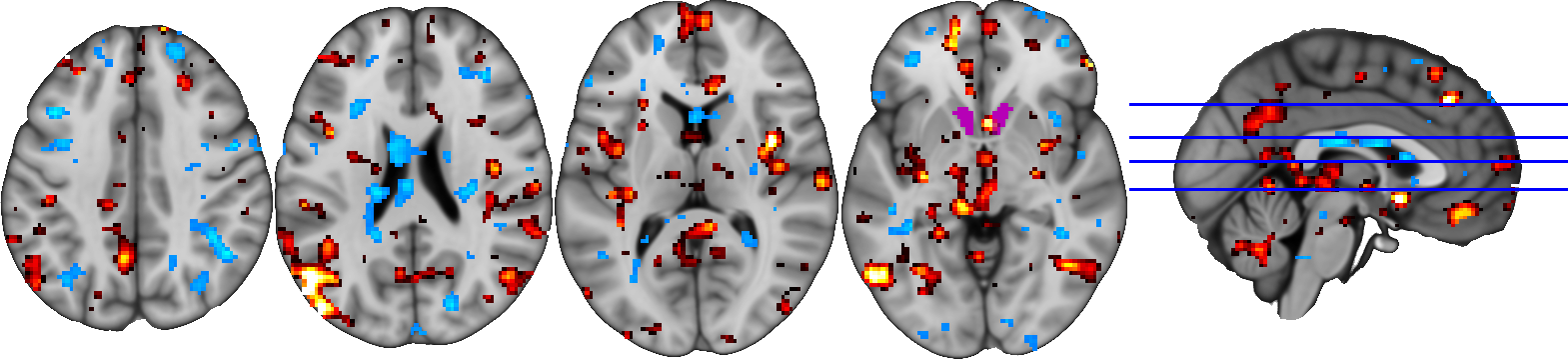


**NAc RSFC (Music>Rest, After)**


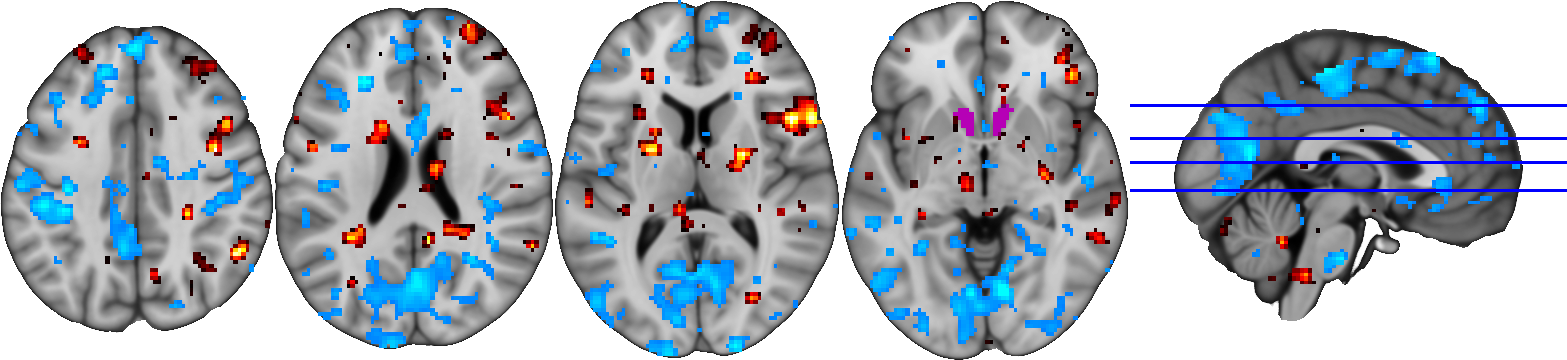


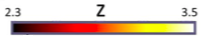


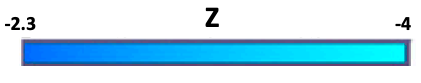


**Figure 1b.** Seed-based functional connectivity (FC) for nucleus accumbens (NAc, displayed in purple) for music > no-music, before treatment (top row) and after treatment (bottom row). Hot colors = increased FC, blue colors = decreased FC. Z>1.6. This lower threshold was chosen for illustration purposes. Left side represents left hemisphere.
